# Correlates to psychological distress in frail older community-dwellers undergoing lockdown during the COVID-19 pandemic

**DOI:** 10.1186/s12877-022-03072-w

**Published:** 2022-06-23

**Authors:** Carmina Castellano-Tejedor, Laura M. Pérez, Luis Soto-Bagaria, Ester Risco, Maria Victoria Mazo, Ana Gómez, Daniel Salvador, Javier Yanguas, María B. Enfedaque, Alessandro Morandi, Mariona Font, Vanessa Davey, Marco Inzitari

**Affiliations:** 1 Parc Sanitari Pere Virgili, Area of Intermediate Care, REFiT BCN Research Group, Gregal 0, 08030 Barcelona, Spain; 2 RE-FiT Barcelona Research Group. Vall d’Hebron Institute of Research and Parc Sanitari Pere Virgili, Barcelona, Spain; 3 GIES Research Group, Basic Psychology Department, Autonomous University of Barcelona, Bellaterra, Spain; 4 Primary Healthcare Center Barceloneta, Atenció Primària Pere Virgili, Barcelona, Spain; 5 Primary Healthcare Center Bordeta-Magòria, Institut Català de La Salut, Barcelona, Spain; 6 Fundació Privada Avismón-Catalunya, Barcelona, Spain; 7 Programa de Mayores. Fundación “La Caixa”, Barcelona, Spain; 8 Institut Català de La Salut, ICS, Barcelona, Spain; 9 Geriatric Unit, IRCCS Istituti Clinici Scientifici Maugeri, Milan, Italy; 10 Department of Clinical Sciences and Community Health, Università Di Milano, Milan, Italy; 11 Department of Medicine, Universitat Autònoma de Barcelona, Barcelona, Spain; 12 Faculty of Health Sciences, Universitat Oberta de Catalunya (UOC), Barcelona, Spain

**Keywords:** COVID-19, Frailty, Ageing, Psychological distress, Lockdown

## Abstract

**Background:**

This study identifies correlates of the lockdown’s psychological distress in frail older community-dwellers (Catalonia, Spain).

**Methods:**

Participants from a community frailty intervention program, with a comprehensive geriatric assessment within the 12-months pre-lockdown and COVID-19 free during the first pandemic wave (March–May 2020), underwent a phone assessment past the lockdown to assess COVID-19-related emotional distress (DME) as well as other sociodemograhic, clinical and psychosocial factors.

**Results:**

Of the 94 frail older adults (age = 82,34 ± 6,12 years; 68,1% women; 38,3% living alone), 84,9% were at risk of experiencing moderate-to-high psychological distress, according to the backward stepwise logistic regression model obtained (χ2 = 47,007, *p* < 0,001, Nagelkerke R^2^ = 0,528), based on the following factors: absence of depressive symptoms before lockdown (OR = 0,12, *p* = 0,014, 95%CI[0,023–0,647]), not carrying out leisure activities during lockdown (OR = 0,257, *p* = 0,023, 95%CI[0,079–0,832]) and currently experiencing high malaise due to COVID-19 situation (OR = 1,504, *p* < 0,001, 95%CI[1,241–1,822]).

**Discussion:**

These findings suggest that it is necessary to favour a prior overall health status and to empower frail older community-dwellers in the use of a broad repertoire of coping strategies in the face of adversity to foster mental health and keep at bay the potential emotional impact of the situation generated by the COVID pandemic.

## Background

COVID-19 is transmitted between humans in close proximity. Thus, besides individual preventive hygiene measures, physical-social distancing is crucial for reducing its spread. By April 2020, most countries worldwide had introduced quarantine measures, travel bans, cancelled social events, and closed public services to contain COVID-19 [24, 53]. Confinement and isolation have been proven to be highly effective in reducing the risk of contracting and spreading COVID-19 [12, 23, 52]. However, previous outbreaks of SARS and MERS showed that such measures could negatively affect some individuals’ mental health, with increases in psychological distress or even psychiatric symptoms linked to stress reactions such as anxiety and depression [9, 28]. Similar mid-to-long term effects have been reported due to the COVID-19 situation [18]. This potentially traumatic stressor might cause or exacerbate psychological distress [7]. High levels of psychological distress indicate impaired mental health and may reflect common mental disorders, like depressive and anxiety disorders.

Multiple factors may trigger increased psychological distress during the pandemic. Importance has been given to the direct effects of isolation, with restrictions on movements and sedentary lifestyles [41] and impoverishment of social contacts [20]. Psychological distress may also arise from the immediate need to adapt to new lifestyles and change day-to-day routines [2, 15]. Also, an increased state of alert due to fear of contagion, grief, or mourning the loss of family members or friends for COVID-19 may undermine wellbeing [19]. All these considerations apply to the general population, and very little information is available for older adults, one of the most vulnerable collectives in society.

So far, data suggest that COVID-19 is associated with particularly unfavorable outcomes when infecting frail older people. Unfortunately, the impact of the COVID-19 pandemic on this population may extend beyond directly related morbidity and deaths to also include a negative influence on pre-existing clinical conditions and geriatric syndromes [13, 38]. The burden of the pandemic on older people is therefore likely underestimated by official statistics, which almost exclusively focus on mortality and the number of infected persons. During the actual COVID-19 pandemic, older adults were specially advised to minimize social contact with people outside their household and remain at home wherever possible. Consequently, it increases the risk of experiencing significant psychological distress in a population already vulnerable to loneliness and social isolation [9, 17, 28]. In line with earlier reports, high levels of depressive disorders and anxiety were observed among older adults, often associated with physical health deterioration and decreased social support [21]. A priori, COVID-19 provides the “perfect storm” for undermining older adults´ mental health. However, specific knowledge of the psychological impact of social distancing measures on frail older individuals is still scarce [1].

From this perspective, this study sought to investigate the impact of lockdown on psychological symptoms (specifically, COVID-related distress) on frail community-dwelling older people who had not been diagnosed with COVID-19 during the first trimester of COVID-19 strict lockdown. Factors that could modulate COVID-19-related distress such as gender increased previous physical and psychosocial vulnerability and negative consequences derived from the pandemic (i.e., loss of a loved one, isolation) were also investigated.

## Methods

### Study population and design

The study population was derived from the + ÀGIL Barcelona project, an ongoing “real-life” intervention program to promote healthy lifestyles [42, 43] in Catalonia, Spain. This program enrols to a longitudinal design study, non-disabled frail older adults presenting with at least one sign of frailty (i.e., slow gait speed, weakness, memory complaints, involuntary weight loss, poor social support). + ÀGIL offers a multicomponent tailored-intervention to slow or revert frailty and promote health-related quality of life. One of the main components of the + ÀGIL Barcelona program is physical activity mediated via a 10-week physical exercise program combining at-home monitored sessions based on the ViviFrail platform [27] with weekly group sessions (one day per week). A physical activity maintenance plan is incorporated, taking advantage of existing resources in the community to boost and support participants to become more active. Promotion of a Mediterranean diet, health education, and optimization of pharmacological therapies are also part of the intervention. After the initial comprehensive geriatric assessment to tailor the intervention, the geriatrician performed follow-ups at 3 and 6 months to revise and refine the intervention as appropriate.  + ÀGIL Barcelona program activity has been continuously running from July 2016 until March 2020 (enrolling up to 245 older persons). Due to the COVID-19 pandemic, the face-to-face assessments were temporarily suspended, and phone follow-ups were carried out instead.  + AGIL Barcelona protocol and preliminary results have been described elsewhere [26, 42, 43].

### Procedure and data collection

In May 2020, at the end of the Spanish lockdown applied by the Spanish Government (7 weeks, from March 14th to May 2nd), participants already included in the + ÀGIL program and waiting for follow-up evaluation were contacted by telephone. To be included in the present study, participants needed to have been previously assessed face-to-face during the 12 months before the lockdown (either at the baseline, 3, and/or 6-month follow-up visit). If the participant could not attend the phone call, a self-identified proxy or caregiver answered the follow-up interview. The interviews lasted around 20 min and were performed by two trained physiotherapist researchers. Out of 117 contacted participants from the + ÀGIL Barcelona program, 107 (91.5%) accepted to answer the phone survey. The remaining cases were not reached or did not want to answer the phone survey. Considering the total included respondents, four participants (3.7%) were excluded from final analyses due to a positive diagnosis of SARS-COV-2 to ensure the studied sample’s homogeneity, and nine participants (8.4%) were excluded from analyses since no data was available regarding the primary outcome variable (psychological distress, assessed with the *Detection of Emotional Distress Scale* [DME]); [32, 33].

### Main outcome variable

Emotional distress was screened with the *Detection of Emotional Distress Scale* (DME); [32, 33], a brief questionnaire consisting of two parts. The first one contains three questions addressed to the patient; self-perceived mood and coping with the situation (numeric scale ranging from 0–10) and a multiple-selection check item recording the presence or the absence of different specific concerns. The second part consists of clinical observation of external signs of emotional distress by the healthcare professional. The DME gives a total score from 0 to 20, consisting of the sum of the direct scores of the answers to the questions concerning mood assessment and how it is handled (first part), higher scores indicating higher emotional distress. According to the previously established cut-off, the total DME score was categorized into mild emotional distress and moderate-severe emotional distress. Questions about concerns or external signs of emotional distress allow healthcare professionals to provide more targeted care. Cronbach’s alpha for this study was 0.83.

### Covariates

Data from the last face-to-face comprehensive geriatric assessment (CGA) pre-lockdown were considered covariates. These included: sociodemographic data (age, gender, living alone, education level), clinical characteristics including *Charlson Comorbidity Index *[14], functional independence for basic activities of daily living (*Barthel index* [0–100 points] [36]) and instrumental activities for daily living (*Lawton index* [0–8 points] [30]), previous diagnosis of cognitive impairment and/or dementia, or positive screening (Minicog range 0–5; < 3 positive screening for cognitive impairment [6, 10]), depressive symptoms (*Yesavage Geriatric Depression Scale* [0–15 points] [25, 54]), physical function (*Short Physical Performance Battery* [SPPB, 0–12 points] [22, 51]), practice of physical activity (*Brief Physical Activity Assessment Tool* [BPAAT, 0–8 points] [44]), frailty status (*Clinical Frailty Scale* (CFS) [45]), self-perceived social loneliness (*Loneliness Scale* [ESTE II], 0–30 points [46]) self-reported health status (*EuroQol-5 Dimension* [EQ-5D] [4]) and self-perceived quality of sleep.

Data collected by semi-structured phone interview during the lockdown included: sociodemographic data (age, gender, cohabitation), support at home, social relations with family or other persons, tools to maintain social contact and frequency, COVID-19 diagnosis on relatives, new onset of acute clinical events, self-reported fatigue, frailty-related symptoms [42, 43], cancelled health visits due to the pandemic and communication with healthcare professionals, functional independence for basic activities of daily living (*Barthel index* [0–100 points] [36]), the practice of physical activity *(Brief Physical Activity Assessment Tool* [BPAAT, 0–8 points] [44]), self-perceived quality of life, self-perceived quality of sleep and activities performed to stay active during the lockdown.

### Statistical analysis

Characteristics of the sample before lockdown are presented as mean values and standard deviation or median values and interquartile range for continuous variables as applicable, and frequency and percentages for categorical variables. According to the total DME score, the primary outcome variable was the categorized psychological distress considering the cut-off ≥ 9 (mild emotional distress vs. moderate-to-severe emotional distress) as recommended by the authors’ of the instrument. Differences among participants with mild and moderate-to-severe psychological distress due to the COVID-19 pandemic were analyzed using the Student’s t-test or the Mann–Whitney U-test and Chi-square test, as appropriate. Variables showing an association with the main outcome variable (*p*-value < 0,05) and those considered clinically relevant or to have a potential influence on the main outcome variable were included in a backward stepwise logistic regression model (emotional distress as dichotomous outcome). This stepwise approach is useful because it reduces the number of predictors, reduces the multicollinearity problem, and is one way to resolve the overfitting. All analyses were performed using SPSS version 20.

### Ethical aspects

The + ÀGIL Barcelona program and study protocol were approved by the Clinical Research Ethics Committee of the *Institut Universitari d’Investigació en Atención Primaria Jordi Gol i Gorina* (reference number 20/048-P) and *Comisión de Ética en la Experimentación Animal y Humana*, *Universitat Autònoma de Barcelon*a (reference number CEEAH 5066). All procedures align with internal ethical regulations and those from the World Medical Association and the Declaration of Helsinki of 1975, with its successive amendments. Before starting the telephone interview, oral informed consent was obtained from all participants after receiving the study’s information and the measures to guarantee their data confidentiality and protection. Previously, written informed consent was obtained from all participants at baseline measures and inclusion, as a part of the + ÀGIL Barcelona program and its research procedures (including face-to-face or telephone interviews). If the participant could not provide such consent, it was obtained from a proxy. All data were treated following the Spanish Organic Law 3/2018 of Protection of personal data. Data confidentiality was ensured at all times using a coding system. Data collected has been exclusively used for the present study, and participation was not economically rewarded.

## Results

Out of 117 contacted participants from + ÀGIL, 107 (91,5%) agreed to answer the phone survey. To ensure the homogeneity of the population, those previously diagnosed with SARS-COVID-19 (*n* = 4), or with incomplete data (*n* = 9), were excluded. Finally, we included in the analyses 94 participants (mean age = 82,34 ± 6,12 years; 68,1% women; 38,3% living alone; mean time since last face-to-face visit = 8,2 ± 3,8 months). There were no significant differences in terms of age, gender, and time since the last face-to-face assessment between those who accepted and those who refused to participate or were excluded from the survey.

Characteristics of the sample before the lockdown due to COVID-19 are displayed in Table 1.

**Table 1:** Pre-lockdown characteristics and association with emotional distress during the COVID-19 lockdown

**Baseline characteristics (pre-lockdown)**	**Included *N* = 94**	**Mild emotional distress during lockdown, *n* = 41**^a^	**Moderate-to-severe emotional distress during lockdown, *n* = 53**^a^	***p*-value**
Age, mean (SD)	82,4 (6,1)	83,2 (5,9)	81,7 (6,3)	0,233
Women, % (n)	68,1 (64)	56,1 (23)	77,4 (41)	**0,028**
Living alone, % (n)	55,3 (52)	61,0 (25)	50,9 (27)	0,332
Education, % (n)				
Illiterate	8,6 (8)	12,2 (5)	5,8 (3)	0,357
Primary school	37,6 (35)	43,9 (18)	32,7 (17)	
Secondary school	39,8 (37)	31,7 (13)	46,2 (24)	
University degree	14,0 (13)	12,2 (5)	15,4 (8)	
Charlson Comorbidity Index, median (IQR)	2 (1–3)	1 (1–3)	2 (1–3)	0,433
Barthel index^b^, median (IQR)	95 (90 – 100)	95 (90 – 100)	95 (90 – 95)	0,185
Lawton index^c^, median (IQR)	5,5 (3–8)	5 (3–8)	6 (3–8)	0,576
Previous diagnosis/positive screening of cognitive impairment^d^, % (n)	33,3 (31)	42,5 (17)	26,4 (14)	0,103
Depressive symptoms (Yesavage GDS-15)^e^, % (n)	21,3 (20)	4,9 (2)	34,0 (18)	**0,001**
Vulnerable and/or any frailty degree (CFS)^f^, % (n)	62,8 (59)	65,9 (27)	60,4 (32)	0,586
Sufficient physical activity^g^, % (n)	60,9 (56)	53,7 (22)	66,7 (34)	0,204
SPPB, mean (SD)	8,5 (2,9)	8,7 (3,1)	8,4 (2,8)	0,662
Gait speed, mean (SD)	0,77 (0,21)	0,79 (0.20)	0,73 (0,22)	0,244
ESTE II total, mean (SD)^h^	12,5 (9–15)	12,1 (4.6)	12,9 (94,8)	0,448
ESTE II–Social support, mean (SD)	3,5 (3,0)	3,1 (2,7)	3,9 (3,1)	0,185
ESTE II–Use of new technologies, mean (SD)	4,1 (1,6)	4,0 (1,5)	4,2 (1,6)	0,437
ESTE II–Self-report social participation, mean (SD)	4,9 (2,4)	5,1 (2,5)	4,7 (2,4)	0,464
Self-reported health status, % (n)				
Low	14,9 (14)	12,2 (5)	17,0 (9)	0,808
Normal or regular	25,5 (24)	26,8 (11)	24,5 (13)	
Good or Excellent	59,6 (56)	61,0 (25)	58,5 (31)	
Self-reported sleep quality, good–excellent, % (n)	48,9 (46)	56,1 (23)	43,4 (23)	0,222

*IQR* Interquartile range, *SD* Standard deviation

Student’s t-test or the Mann–Whitney U-test were used for continuous variables as appropriate and Chi-square test for categorical

^a^Detection of Emotional Distress Scale (DME scale): scoring range from 0–20 (≥ 9 moderate-to-severe emotional distress)

^b^Independence for activities of daily living, Barthel index: scoring range from 0–100

^c^Independence for instrumental activities of daily living, Lawton index: scoring range from 0–8

^d^Previous diagnosis of cognitive impairment or dementia, or positive screening performed with Minicog: scoring range 0–5 points (< 3 positive screening for cognitive impairment)

^e^Geriatric Depression Scale Yesavage (GDS-15): scoring range from 0–15 points (> 5 points: probable depression)

^f^Clinical Frailty Scale (CFS): scoring range from 1–9 points (1–3: very fit-fit, managing well; 4–6: mild-to-moderate frailty; 7–9: severe frailty-to-terminally ill)

^g^Brief Physical Activity Assessment Tool (BPAAT): scoring range from 0–8 (≥ 4 points: sufficient active, 0–3: insufficient active)

^h^Social Loneliness Scale (ESTE-II): scoring range 0–30 (≥ 11 points: moderate-to-high social loneliness)

During the COVID-19 lockdown, malaise directly related to the COVID pandemic situation was assessed by means of the distress thermometer, and results were medium-to-high (M = 5,57 ± 3,37, range 0–10) with approximately one-third of the sample scoring ≥ 8 points (*n* = 33, 35%). Psychological distress assessed through the DME assessment tool (total score) was also medium-to-high (M = 8,55 ± 4,30, range 0–18), with half of the sample (*n* = 53, 56,4%) scoring ≥ 9 points (DME cut-off). Higher psychological distress during the lockdown was associated with the female gender (*p* = 0,028) and the presence of depressive symptoms (*p* = 0,001), with a higher proportion of women and individuals experiencing depressive symptoms within the group of moderate-to-severe emotional distress. Despite not reaching statistical significance, this group of individuals were also a subsample characterized by living alone, being older, having a cognitive impairment and displaying a frailer and more sedentary profile.

During the lockdown, individuals showing a profile of moderate-to-severe psychological distress due to the COVID-19 situation tended to use coping strategies based on disengagement and distraction, including leisure activities such as painting, crafts, table games, urban gardening (*p* = 0,012), watching TV, or listening to music (*p* = 0,029). Also, these individuals reported significantly lower quality of life (*p* = 0,033), were more likely to have concerns for their family wellbeing relating to COVID-19 (*p* = 0,011), and experienced more discomfort (*p* < 0,001) and moderate difficulties in coping with the situation created by the COVID-19 pandemic (*p* < 0,001), when compared to mild or non-distressed individuals (see Table 2).

**Table 2:** Description of characteristics of the sample during the lockdown due to COVID-19

**Characteristics**	**Included *N* = 94**	**Mild emotional Distress during lockdown, *n* = 41**^a^	**Moderate-to-severe emotional distress during lockdown, *n* = 53**^a^	***p*-value**
Living alone, % (n)	38,3 (36)	34,2 (14)	41,5 (22)	0,466
Who lives at home, % (n)				
Family	53,2 (50)	58,5 (24)	49,1 (26)	0,718
Formal caregiver	4,3 (4)	2,4 (1)	5,7 (3)	
Family and formal caregiver	4,3 (4)	4,9 (2)	3,8 (2)	
Social contact maintained by, % (n):				
Phone	77,0 (67)	66,7 (24)	84,3 (43)	0,154
Video-call	9,2 (8)	13,9 (5)	5,9 (3)	
Phone and Video-call	13,8 (12)	19,4 (7)	9,8 (5)	
Social contact different than family, % (n)	46,8 (44)	36,6 (15)	54,7 (29)	0,081
Frequency of social contact, % (n)				
Daily	84,0 (79)	82,9 (34)	84,9 (45)	0,397
Once/twice a week	11,7 (11)	9,8 (4)	13,2 (7)	
No contact	4,3 (4)	7,3 (3)	1,9 (1)	
Received support for housework, % (n)	40,4 (38)	41,5 (17)	39,6 (21)	0,857
Received help with groceries and medication, % (n)	100,0 (94)	100,0 (41,0)	100,0 (53)	
Activities to stay active during lockdown^b^, % (n)				
Housework	47,9 (45)	43,9 (18)	50,9 (27)	0,498
Leisure activities^c^	36,2 (34)	22,0 (9)	47,2 (25)	**0,012**
Music/ TV	70,2 (66)	58,5 (24)	79,3 (42)	**0,029**
Provide care	5,3 (5)	2,4 (1)	7,6 (5)	0,274
Reading	27,6 (26)	26,8 (11)	28,3 (15)	0,874
Social contact	10,6 (10)	14,6 (6)	7,6 (4)	0,269
Use of technology	5,3 (5)	7,3 (3)	3,8 (2)	0,448
Self-reported health status, % (n)				
Low	6,4 (6)	0,0 (0)	11,3 (6)	**0,033**
Normal or regular	66,0 (62)	63,4 (26)	67,9 (36)	
Good or Excellent	27,7 (26)	36,6 (15)	20,8 (11)	
Self-reported sleep quality, good-excellent^d^, % (n)	50,0 (47)	61,0 (25)	41,5 (22)	0,061
Family member got COVID-19, % (n)	8,5 (8)	2,4 (1)	13,2 (7)	0,064
Relative died due to COVID-19, % (n)	2,1 (2)	0,0 (0)	3,8 (2)	0,209
Moderate discomfort due to COVID-19 situation^e^, % (n)	45,7 (43)	17,1 (7)	67,9 (36)	** < 0,001**
Main concern during COVID (item 2 DME), % (n)				
Economic situation	9,6 (9)	7,3 (3)	11,3 (6)	0,513
Family situation	30,9 (29)	17,1 (7)	41,5 (22)	**0,011**
Emotional	5,3 (5)	7,3 (3)	3,8 (2)	0,448
Health-related	6,4 (6)	4,9 (2)	7,6 (4)	0,600
Others (not specified)	9,6 (9)	7,3 (3)	11,3 (6)	0,513
No concerns	37,2 (35)	53,7 (22)	24,5 (13)	**0,004**
Moderate difficulty coping with the COVID-19 situation (item 3 DME)^f^ % (n)	33,0 (31)	0,0 (0)	58,5 (31)	** < 0,001**

^a^Detection of Emotional Distress Scale (DME scale): scoring range from 0–20 (≥ 9 moderate-to-severe emotional distress)

^b^Not mutually exclusive

^c^This category includes: painting, crafts, table games, urban gardening

^d^Self-reported sleep quality: excellent, good, regular, bad, very bad

^e^Discomfort due to COVID-19 (distress thermometer item from the DME): scoring range from 0–10 (≥ 7 points: moderate discomfort)

^f^Difficulty coping with COVID-19 situation (item 3 from the DME): scoring range from 0–10 (≥ 7 points: moderate difficulty coping)

A statistically significant model (χ^2^ = 47,007, *p* < 0,001, Nagelkerke R^2^ = 0,528) was obtained, correctly classifying 84,9% of individuals at risk of experiencing moderate-to-high psychological distress, based on the following factors: the absence of depressive symptoms before lockdown, not carrying out any type of the leisure activities during the lockdown, such as painting, crafts, table games, urban gardening or listening to music or watching TV, and expressing high malaise due to COVID situation (see Table 3).

**Table 3:** Association with moderate emotional distress during the COVID-19 lockdown. Multivariable logistic regression analysis

	**OR Exp (B)**	**95%CI for Exp (B)**	***p*-value**
Depressive symptoms^a^ (pre-lockdown)	0,121	0,023—0,647	**0,014**
Leisure activities^b^ (during lockdown)	0,257	0,079—0,832	**0,023**
Malaise due to COVID-19 situation (during lockdown)	1,504	1,241—1,822	** < 0,001**

^a^Assessed by means of the Geriatric Depression Scale Yesavage (GDS-15)

^b^Leisure activities as a specific type of coping to stay active during lockdown

## Discussion

In our sample, the general psychological impact of the lockdown in older frail, pre-disabled community-dwellers non affected directly by COVID-19 was medium-to-high. One-third of them expressed significant malaise due to the COVID situation (distress thermometer), and half of the sample reached the cut-off point in the DME assessment tool, indicating clinically significant emotional distress. This study also revealed that factors identifying at-risk individuals to suffer moderate-to-high psychological impact are related to previous mental health status, current experience and intrapersonal resources to cope with the situation imposed by the pandemic.

Our results are in line with previous research that has pointed out that women are more prone to suffer from psychological distress during strict lockdown measures related to health crises. This was already documented during the equine or the H1N1 influenza outbreak, and preliminary evidence related to the COVID-19 pandemic also points in this direction [11, 35]. A possible explanation for this is that, regardless of age, in Western cultures, women present a more externalizing pattern of reporting feelings and emotions, their degree of emotional awareness is usually higher, and they tend to share more openly their experiences and the possible impact of potentially traumatic experiences than men [48]. Another explanation for these results may be that the prevalence of depression and anxiety is generally higher in women [3, 47].

Most frequently reported concerns during the COVID-19 pandemic were those related to the health status of a family member and close relatives, coinciding with most research in this field [11, 35, 50]. In this line, family situation concerns were associated with moderate-to-high psychological distress. Most reports have pointed out that the general state of worries was high at the beginning of the pandemic and tended to decrease steadily throughout the subsequent months; with no significant effect depending on the different official announcements and interventions [50]. Despite not having evidence in this sense in our study, it is also possible that women suffer from a greater care burden due to the increased need for care both outside and inside the home during lockdown (i.e., husband and/or grandchildren) [34, 37].

Faced with the experience of a potentially overwhelming and traumatic situation, people put in place a series of coping strategies and more or less conscious defence mechanisms to help to overcome perceived difficulties and/or threats. Our sample revealed a disengaged coping style to self-regulated distress generated by the pandemic and lockdown situations. In this sense, the most distressed individuals report greater use of distraction such as leisure activities (i.e., painting, crafts, table games, urban gardening) and watching TV or listening to music to mitigate their psychological impact, compared to those individuals experiencing mild emotional distress. These results are in line with previous research pointing out that strategies reflecting disengagement copings, such as passive reaction pattern, palliative reaction, and avoidance, were associated with less perceived control and negatively associated with psychological wellbeing [16]. In this sense, it is important to highlight that all individuals reporting moderate-to-high difficulties coping with the situation are within the moderate-to-high distress individuals’ group, with no cases expressing significant difficulties to cope in the mild-distressed individuals’ group.

A strong relationship between self-perceived general health status and mental health has been noted in several studies [5]. When analysing differences in self-reported health of the participants, a higher percentage of individuals reporting low health status was observed among those in the moderate-to-high distress group. These differences reached statistical significance when they were reassessed during confinement.

Our study reveals several factors that could serve to identify and characterize this sample of at-risk individuals to suffer moderate-to-high distress due to the COVID-19 situation. Among frail community older adults not diagnosed with COVID during the first trimester of strict lockdown, those not carrying out any leisure activities during the lockdown, and not having experienced depressive symptoms before this period but expressing current significant malaise due to COVID-19 situation are those at higher risk of moderate-to-high psychological impact.

As some research has demonstrated, there is a clear interplay between physical activities and psychological factors in the general population and, specifically, among older adults [39]. Evidence has shown that regular physical activities help develop self-efficacy and bring changes in the perception of one’s health and happiness, thereby reducing depression and increasing and broadening coping resources. These associations seem relevant for those with previous psychopathological symptoms and/or diagnosis [7] but also, for individuals who have not faced severe situations throughout their lives and, therefore, might not have a vast repertoire of coping strategies or might have low self-efficacy to deal with the possible impact that a serious situation can imply, such as that generated by the COVID-19. Due to restrictions on outside activities during the social distancing and quarantine period, it seems crucial to make time for healthy habits combined with leisure and enjoyable activities not only based on disengagement (e.g., indoor physical activities such as stretching, gymnastics, yoga, hula hoop, and dumbbell exercise).

Contrary to what was expected, having suffered the loss of a loved one and living alone was not related to higher odds of suffering from psychological distress. This might be due to the low prevalence of grieving individuals in our sample and the long time they were already living alone before the COVID-19 pandemic. Also, there were no significant differences in the means or frequency of social contact between individuals that experienced moderate to severe emotional distress and individuals whose emotional distress was only mild. Studies have shown that older adults [49], as well as younger adults (between 18–35 years of age) [29], reported high levels of loneliness after social distancing measures were implemented. However, some research suggests that older adults may be more resilient to the negative effects on mental health [40] and younger adults might be at greater risk for heightened loneliness during the pandemic [8]. In our study, perhaps due to the low variability in the age range included in the study sample, these associations could not be found.

In light of these results, our findings suggest that it is necessary to favour a prior overall health status and, at the same time, to empower individuals in the use of a broad repertoire of coping strategies in the face of adversity to foster mental health and make it easier to keep concerns at bay.

This study has different limitations. Data were mainly collected through self-report measures and semi-structured interviews by phone, which presents a possible bias in the responses and the one-sided view of the participants’ psychological conditions. More objective, unbiased measures are recommended in similar future studies addressing the older adults’ mental health. However, we consider this research has implemented the best possible methodology to address the study objectives in such specific complex circumstances imposed by the COVID-19. Also, we used a small convenience sample of individuals already recruited for a previous project, and creating study subsamples to carry out different analyses, makes it difficult to generalize these results to different populations within the same age group and therefore, could limit the statistical power.

Despite these limitations, this work has different strengths. First of all, it provides information about a “real world” cohort of very well-characterized patients since they are part of a larger project,  + AGIL BCN [42, 43]. Therefore, a wide array of pre-and post-lockdown health-realted pre-pandemic measures are available, which adds a valuable standpoint to analyze the psychological impact of the pandemic on this vulnerable and understudied sample population. Secondly and, in the same line, we believe this research adds to the existing knowledge by providing evidence on the demographic, clinical and psychographic COVID-19 related variables that might influence older adults mental health. The COVID-19 situation called for immediate measures that deal with the profound impact of confinement, quarantine and the uncertainty that the general population suffered, and particularly, at-risk vulnerable groups. All these knowledge could help in future heath crises. However, more studies with prospective longitudinal designs in this line and with this specific population are still necessary, to further identify the relevant protective factors for older adults’ mental health and to validate and refine factors that entail the most significant risk [31]. We believe that efforts to protect mental health are equally important as efforts to physically prevent and treat COVID-19 or other pandemics, especially among the older adults. Analyzing, proposing, and implementing practical psychological and mental treatment strategies for this sample population must be a priority. Thus, we strongly encourage further research in this matter, which is now essential to tackle the emotional and psychological impact associated with the pandemic. That will be the key to protect and ensure older adults mental health in similar and possible future scenarios.

## Data Availability

The datasets generated and/or analysed during the current study are not publicly available due to the sensitive nature of the personal health data collected from a vulnerable population and privacy and confidentiality reasons, but might be partially available from the corresponding author on reasonable request.

## Abbreviations

BPAAT: Brief Physical Activity Assessment Tool
CFS: Clinical Frailty Scale
CGA: Comprehensive Geriatric Assessment
DME: Detection of Emotional Distress Scale
ESTE II: Self-perceived Social Loneliness Scale
EQ-5D: European Quality of Life Self-reported measure-5 dimensions
GDS-15: Geriatric Depression Scale Yesavage-15 items
SPPB: Short Physical Performance Battery
